# Aerosolization effects on coronavirus infectivity

**DOI:** 10.3389/fmicb.2026.1764341

**Published:** 2026-02-20

**Authors:** Meiyi Zhang, Gabriel L. Hamer, Maria D. King

**Affiliations:** 1 Department of Biological and Agricultural Engineering, Texas A&M University, College Station, TX, United States; 2 Department of Entomology, Texas A&M University, College Station, TX, United States

**Keywords:** aerosolization, aerosols, bovine coronavirus, nebulization, SARS-CoV-2, virus infectivity

## Abstract

Understanding the airborne persistence of coronaviruses is critical for effective infection control, yet the effects of aerosolization and airborne suspension on viral infectivity remain poorly defined. In this study, we used bovine coronavirus (BCoV) as a surrogate for human *Betacoronaviruses* to evaluate how nebulization, aerosolization time, and continuous air mixing affect virus viability and RNA persistence. BCoV suspensions were aerosolized in a sealed, propeller-mixed chamber using a Collison nebulizer for 5, 10, 15, 30, and 45 min. Aerosols were collected using a Wetted Wall Cyclone (WWC), and post-nebulization suspension from the nebulizer reservoir and original stock were analyzed. Infectivity was quantified by TCID_50_ assay on MDBK cells, and viral RNA was measured by qRT-PCR. Stock and nebulized suspensions retained stable infectivity, indicating that the mechanical forces of nebulization did not impair viral viability. In contrast, WWC-collected aerosols showed a time-dependent infectivity decline. Viral RNA in aerosols remained comparatively stable, whereas RNA levels in the nebulizer reservoir dropped during the first 5 min of nebulization and then remained constant. Temperature and relative humidity in the chamber during the tests showed only minor fluctuations. These findings showed minimal loss of BCoV viability in suspensions during nebulization and significant inactivation at prolonged air mixing, while RNA levels persist. The pronounced infectivity loss under continuous air mixing highlights the role of mechanical stresses in compromising airborne coronavirus viability, a factor directly relevant to indoor environments where HVAC systems and fans are commonly used. The findings of this study help inform risk mitigation strategies in real-world settings.

## Introduction

Coronaviruses are a family of enveloped positive-sense single-stranded RNA viruses that can cause respiratory diseases in a variety of animal hosts, including humans (Farhud et al., 2022). Over the past two decades, three highly pathogenic human coronaviruses - Severe acute respiratory syndrome coronavirus (SARS-CoV), Middle East Respiratory Syndrome coronavirus (MERS-CoV), and most recently severe acute respiratory syndrome coronavirus 2 (SARS-CoV-2) - have triggered global epidemics (Peeri et al., 2020). The emergence of these high-consequence respiratory viruses has spurred global efforts to understand the environmental stability and transmission dynamics of coronaviruses. Bovine coronavirus (BCoV), a member of the *Betacoronavirus* genus alongside SARS-CoV, MERS-CoV, and SARS-CoV-2, has been widely used as a surrogate for human coronaviruses in environmental studies due to their structural and genomic similarities, and lower pathogenic risk (Kimbrell et al., 2024; Islam et al., 2021; Watanabe et al., 2022; Cantu et al., 2023). Although differences in spike protein sequence and lipid envelope composition exist across coronaviruses, recent studies have directly evaluated BCoV alongside SARS-CoV-2 on common surfaces and in engineered wastewater treatment or concentration systems, demonstrating similar trends in viral decay and genome persistence under comparable conditions (Watanabe et al., 2022; Guérin-Rechdaoui et al., 2022; LaTurner et al., 2021; Graham et al., 2021). Similar to other enveloped RNA viruses, the survival and persistence of BCoV is heavily influenced by environmental factors such as temperature and relative humidity (Wei et al., 2022). A study found a significant decline of SARS-CoV-2 viability at elevated temperatures, with a major loss of infectivity at 40 °C compared to room temperature, and complete inactivation at 55 °C (Harapan et al., 2022). Several other studies also supported this trend, which observed reduced persistence and higher decay rate of infectious viruses at increased temperatures (Dabisch et al., 2021; Sharma et al., 2021; Riddell et al., 2020; Zhang et al., 2022). Relative humidity also plays an important role, however with a more complex effect. Several groups have reported rapid loss of virus viability at higher relative humidity (Chan et al., 2011; Biryukov et al., 2020; van Doremalen et al., 2013), whereas Casanova et al. demonstrated the relationship between virus inactivation and relative humidity being non-monotonic as the virus survival rate was higher at low (20%) and high (80%) relative humidity, compared to that at moderate (50%) relative humidity (Casanova et al., 2010). Besides the effects of temperature and relative humidity, extensive work has detailed coronavirus survival on different types of common surfaces, ranging from non-porous surfaces such as plastic and metals to porous surfaces such as fabrics and paper, and in liquid media (Watanabe et al., 2022; Ren et al., 2020; Aboubakr et al., 2021; Baig et al., 2022). As many coronaviruses are known to transmit through the airborne route, a number of researchers have investigated the infectivity of aerosolized coronaviruses under controlled environmental conditions such as temperature, relative humidity, sunlight, droplet size, and suspension media conditions, despite the challenges involved in capturing viable virus and performing infectivity assays (Dabisch et al., 2021; Doremalen et al., 2020; Smither et al., 2020; Schuit et al., 2020; Ijaz et al., 1985; Pyankov et al., 2018). These efforts highlight the ongoing need for systematic evaluation of the effects of aerosolization and the associated environmental stressors on coronavirus infectivity, an important yet underexplored parameter for understanding transmission risk and persistence of viral aerosols in real-world indoor settings.

The bioaerosol generation and aerosolization process itself imposes mechanical stresses such as shear, impaction, and turbulence on bioaerosols that can compromise their microbial integrity (Smith et al., 2025; Smith and King, 2023). Such mechanical forces are not unique to laboratory settings. In fact, the process of aerosolization is similar to air mixing and circulation generated by fans or the Heating, Ventilation, and Air Conditioning (HVAC) systems commonly present in homes, hospitals, schools, and industrial facilities (Thornton et al., 2022). The use of a fan or high-velocity airflow to mix air in enclosed environments can subject airborne particles to shear stress and impact, which can cause physical damages to the microbes (Redmann et al., 2022). The application to mix large volumes of air is frequently found in critical infrastructures such as meat processing plants, which emerged as hotspots for COVID-19 outbreaks, whose environments are characterized by high indoor ventilation, low temperature, enclosed spaces, and vigorous air circulation (Herstein et al., 2021; Pokora et al., 2021; Zhang and King, 2023). These real-world hotspots highlight the importance of understanding how airborne viruses respond not only to environmental conditions, but also to the stresses imposed during air mixing. Our group has previously shown that aerosolization-induced mechanical stress can alter bacterial physiology by promoting lipid aggregation, upregulating antibiotic resistance genes, and inducing dormancy response (Smith et al., 2025; Smith and King, 2023; Smith and King, 2022). Analogous effects on viral particles remain unexplored. While viruses cannot actively respond to stress in the same way as bacteria, the structural integrity of their envelope and capsid may be compromised during aerosolization or turbulent airborne transport, potentially affecting infectivity.

To address this research gap, we aim to investigate how aerosolization and air mixing affect coronavirus infectivity using BCoV as a surrogate for human coronaviruses. Rather than implicating direct equivalence to SARS-CoV-2, this study uses BCoV to investigate mechanisms governing coronavirus stability during aerosolization and airborne residence, with relevance to indoor environments where mechanical air movement, ventilation, and mixing are common. It is part of a broader research effort to characterize how environmental stressors including aerosolization and airflow entrainment affect airborne bacteria and viruses. By comparing infectious titers and gene copy numbers of bioaerosol samples collected at different time intervals of aerosolization and air mixing, our goal is to better understand the influence of these mechanical stresses on airborne coronavirus survival, especially under conditions that mimic fan-driven or HVAC-mediated aerosol dispersion in real-world indoor environments.

## Materials and methods

### Experimental setup

The experimental setup follows a configuration adapted from our previous study (Smith and King, 2022). In a Biosafety Level 2 laboratory, BCoV was aerosolized with a 6-jet Collison nebulizer (BGI, Waltham, MA) into a sealed and insulated chamber (27 L; polypropylene and polyethylene). The chamber was connected to a Wetted Wall Cyclone (WWC) to collect the viral aerosols, and connected to the nebulizer inlet via 1″ diameter vinyl tubing. To consistently mix the air inside the chamber during the aerosolization process, a VOS 16 Propeller and Controller (VWR International, Radnor, PA) was placed at the center of the chamber to maintain a constant airflow at 80 L/min volumetric flow rate and 0.7 m/s air velocity, as verified by an anemometer (TSI Inc., Burnsville, MN). The airflow rate, propeller speed, and chamber geometry used here were identical to those previously characterized using Aerodynamic Particle Sizer (APS) measurements and computational fluid dynamics (CFD) modeling, which demonstrated uniform aerosol mixing, stable particle residence times, and reproducible velocity fields within the chamber under identical operating conditions (Smith et al., 2025). Our prior study showed aerosol particle diameters spanning approximately 0.5 to 7 μm across 5 to 45 min aerosolization periods, with no evidence of significant stagnation or uneven spatial distribution. A HOBO data logger (Onset, Bourne, MA, USA) was placed inside the chamber throughout the experiment to record the temperature and relative humidity in real time. Testing was conducted at room temperature between 22 °C to 25 °C, while the relative humidity increased from 55 to 70% due to nebulization during the longer dissemination periods. To eliminate contamination, all equipment was sterilized by autoclaving or disinfecting with 100% isopropanol and 5% bleach prior to each test. During the testing, all equipment was placed in a biosafety cabinet with continuous airflow to eliminate contamination from the surrounding environment. To confirm the absence of background contamination, a control test was performed with aerosolizing sterile water under identical conditions, and no virus was detected in these blanks.

### Aerosolization and collection

The 6-jet Collison Nebulizer (BGI, Waltham, MA) was filled with 30 mL of BCoV resuspension in 10% Phosphate Buffer Saline (PBS) buffer at pH 7.4 to generate viral aerosols into the sealed chamber. Bovine Coronavirus (BCoV), Mebus Catalog No. NR-445 stock was obtained from BEI Resources (American Type Culture Collection (ATCC), Manassas, VA). The nebulizer was operated at 20 psi for 5, 10, 15, 30 or 45 min depending on the test condition. During aerosolization, a centrally mounted propeller maintained air mixing inside the chamber at a speed corresponding to 0.7 m/s linear air velocity in a ventilated room with heating and air conditioning. Following nebulization and aerosolization, airborne viruses were collected into sterile autoclaved Milli-Q (MQ) water using the WWC bioaerosol collector operated at 100 L/min air inflow rate. Prior to each test, the WWC collector was cleaned by running two cycles of 5% bleach for 1 min, 70% isopropanol for 4 min, and sterile autoclaved MQ water for 10 min, which is a standard disinfecting procedure previously established for WWC operation (King and McFarland, 2012; Zhang et al., 2025). Bioaerosol sampling technologies differ substantially in collection efficiency and viability preservation (Haig et al., 2016; McNeill, 2022). Prior work from our group on the WWC collector demonstrated high recovery efficiency for aerosolized nanoparticles and nano- and micro- size bioaerosols while maintaining culturability and nucleic acid integrity, supporting its suitability for assessing airborne infectivity in the present study (King and McFarland, 2012; Lauterbach et al., 2018; Meng et al., 2014).

### Virus infectivity assay

Titration of infectious virus recovered from the nebulized stock or in the MQ water of the WWC-collected bioaerosol samples was performed using the end-point dilution assay to obtain the 50% tissue culture infectious dose (TCID_50_) in Madin–Darby bovine kidney (MDBK) cells. MDBK cells were cultured in Minimum Essential Medium Eagle (MEME; Corning, Manassas, VA) supplemented with 5% fetal bovine serum (FBS; Avantor, Radnor, PA) and 1% antibiotic–antimycotic (Thermo Fisher Scientific, Waltham, MA). Cells were maintained at 37 °C with 5% CO_2_ and seeded into 96-well flat-bottom tissue culture plates (Corning, Manassas, VA) one day prior to infection to allow formation of a confluent monolayer.

Virus infectivity was assessed by the end-point dilution assay following a previously established method (Watanabe et al., 2022). Nebulized stock samples were serially diluted 1:10 to 1:10^7^ in serum-free MEM, and aerosol samples were diluted 1:2 to 1:128 before inoculation onto MDBK cell monolayers in 96-well plates. The culture was incubated for 6 days at 37 °C with 5% CO_2_. Virus-induced cytopathic effect (CPE) was monitored daily and recorded based on characteristic cell rounding and detachment. TCID_50_ values were calculated based on CPE observations using the Reed-Muench method (Reed and Muench, 1938).

### Virus quantification by qRT-PCR

Quantification of BCoV in the same MQ water of the WWC-collected bioaerosol and nebulized stock was conducted using quantitative reverse transcription-polymerase chain reaction (qRT-PCR) following a previously described method (Baig et al., 2022). Briefly, virus sample was combined with a reaction mixture containing 1 μL of the broadly reactive, CoV RNA-dependent RNA polymerase (RdRp) based 100 μM forward primer IN-2 (5’-GGGTTGGGACTATCCTAAGTGTGA-3′), 1 μL of 100 μM reverse primer IN-4 (5’-TAACACACAACICCATCATCA-3′) (Bellini, 2003), and 5 μL of Power SYBR Green master mix (Applied Biosystems, Foster City, CA). qRT-PCR was carried out on the QuantStudio 3 Real-Time PCR System (Applied Biosystems, Waltham, MA) using the following cycling conditions: 48 °C for 10 min, 95 °C for 10 min, followed by 40 cycles of 95 °C for 15 s and 60 °C for 60 s. Samples were considered positive if amplification was achieved within 40 cycle threshold (Ct < 40). Ct values of positive samples were converted to Gene Copy Number (GCN) per mL based on a standard curve shown in Supplementary Figure S1.

### Statistical analysis

All experiments were performed with three replicates, and results are presented as mean ± standard deviation. Graphing and statistical analyses were conducted using GraphPad Prism (GraphPad Software, San Diego, CA). To assess the effect of nebulization or aerosolization duration on infectious virus titer and viral GCN, non-parametric one-way analysis of variance (ANOVA) using the Kruskal–Wallis test was performed to test the statistical significance among multiple groups, followed by *post hoc* pairwise comparisons using Dunn’s multiple-comparison procedure. A *p*-value less than 0.05 (*p* < 0.05) was considered statistically significant for all comparisons.

## Results

**Figure 1:**
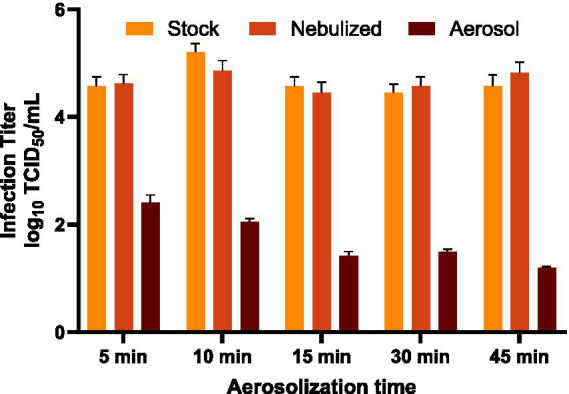
Infectious titers of BCoV in stock suspension, residual nebulized suspension, and WWC-collected aerosols after 5, 10, 15, 30, or 45 min aerosolization along with air mixing. The infectious titers were measured as log_10_ TCID_50_/mL in triplicate after inoculated on MDBK cell culture in 96-well plates and CPE was observed. The limit of detection (LoD) was 1.15 log_10_ TCID_50_/mL for the end-point dilution assay.

BCoV was nebulized by a Collison nebulizer into a sealed, propeller-mixed chamber for 5, 10, 15, 30, and 45 min, after which the virus aerosols were collected using a WWC sampler. Residual liquid remaining in the nebulizer reservoir after each run was also collected, as this sample represents the virus that underwent mechanical nebulization but remained in liquid suspension rather than entering the aerosol phase. This allows comparison between the effects of nebulization on the liquid suspension and the effects of aerosolization plus airborne residence time on virus infectivity. Residual nebulizer liquid (nebulized virus suspension) was sampled alongside the original stock. Infectious titers expressed as log_10_ TCID_50_/mL for stock, nebulized suspensions, and aerosol samples after 5, 10, 15, 30, and 45 min aerosolization are presented in Figure 1. Infectious titers of the stock remained consistent across all runs, ranging from 4.45 to 5.20 log_10_ TCID_50_/mL. Nebulized suspensions exhibited similarly stable infectivity, having a lowest infectious titer of 4.45 log_10_ TCID_50_/mL and highest infectious titer of 4.86 log_10_ TCID_50_/mL. When comparing the stock and nebulized virus suspensions at each aerosolization time point, the nebulized samples had slightly higher infectious titers at 5, 30, and 45 min, and lower infectious titer at 10 and 15 min, although the difference was not statistically significant (*p* = 0.068). In contrast, WWC-collected aerosols showed markedly reduced titers (1.19 to 2.40 log_10_ TCID_50_/mL), falling well below stock and nebulized suspension levels and declining significantly with longer aerosolization (*p* < 0.0001). This suggests that while the viral infectivity was preserved in the liquid resuspension after nebulization, aerosolization under continuous air mixing imposes stresses that progressively inactivate the viruses.

**Figure 2:**
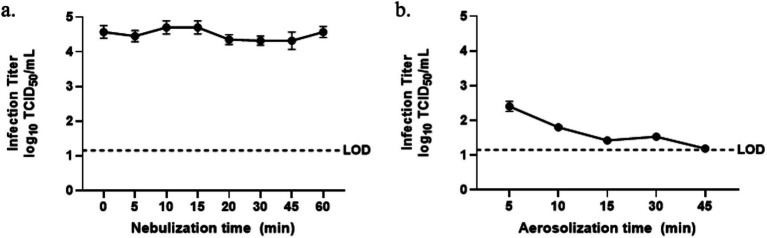
Infectious titers of BCoV **(A)** for the virus suspension in the nebulizer after 0, 5, 10, 15, 20, 30, 45, and 60 min nebulization, and **(B)** for the WWC-collected aerosols after 5, 10, 15, 30, and 45 min aerosolization along with air mixing, measured as log_10_ TCID_50_/mL. The dotted line indicates the assay LoD at 1.15 log_10_ TCID_50_/mL.

To assess whether the process of nebulization alone affects BCoV infectivity in the liquid form, infectious titer of the suspension remaining in the nebulizer after 0 (stock), 5, 10, 15, 20, 30, 45, and 60 min of continuous nebulization is shown in Figure 2A. Infectivity fluctuated modestly between 4.32 and 4.70 log_10_ TCID_50_/mL without a consistent time-dependent trend. One-way ANOVA with *post hoc* comparisons confirmed no significant differences across all tested durations (*p* ≥ 0.22 for all comparisons). The infectivity of the virus aerosol samples collected after 5, 10, 15, 30, and 45 min chamber dispersion was evaluated for the effect of aerosolization and constant air mixing (Figure 2B). The infectivity declined from 2.40 log_10_ TCID_50_/mL at the shortest aerosolization duration tested of 5 min to 1.19 log_10_ TCID_50_/mL at the longest duration of 45 min. After consistently decreased to 1.42 log_10_ TCID_50_/mL at 15 min, the infectivity plateaued to 1.53 log_10_ TCID_50_/mL at 30 min, followed by a slower decrease to 1.19 log_10_ TCID_50_/mL at 45 min. One-way ANOVA indicated a significant overall effect of aerosolization time on virus infectivity (p < 0.0001), and Dunn’s multiple comparisons revealed a significant difference between 5 min and 45 min (*p* = 0.0098). The lowest measured titer approached the assay LoD of 1.15 log_10_ TCID_50_/mL, underscoring that extended aerosol suspension under mixing conditions substantially diminishes viable virus recovery.

**Figure 3:**
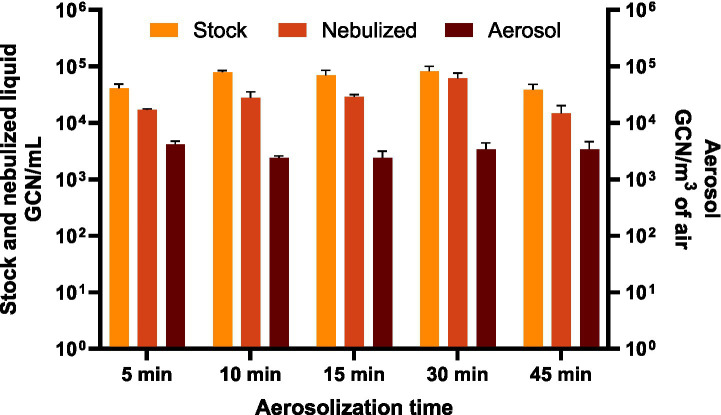
BCoV gene copy numbers per unit volume (GCN/mL) for the stock and nebulized samples, and GCN/m^3^ of air for the aerosol samples collected at 5, 10, 15, 30, and 45 min aerosolization durations.

BCoV RNA levels were quantified in the stock, nebulized, and aerosol samples across different aerosolization durations using qRT-PCR (Figure 3). The virus in the stock suspensions used for aerosolization were relatively consistent, ranging from 3.85 × 10^4^ GCN/mL to 8.22 × 10^4^ GCN/mL. Similarly, the corresponding nebulized samples contained lower levels of BCoV between 1.46 × 10^4^ GCN/mL and 6.12 × 10^4^ GCN/mL. The viral RNA content in the nebulized samples was consistently lower than in the corresponding stock samples for each aerosolization duration tested. A paired t-test confirmed this reduction to be statistically significant (*p* = 0.01), suggesting that the nebulization process itself contributes to a measurable loss or degradation of viral RNA. This decline in RNA concentration may reflect shear stress during nebulization, partial inactivation by impaction, or adsorption to internal surfaces of the nebulizer. The viral RNA levels in aerosol samples collected by the WWC were relatively stable across all time points (*p* > 0.17), ranging from 2.41 × 10^3^ to 4.19 × 10^3^ GCN/m^3^ of air. This limited variability suggests that while viral RNA is recoverable after aerosolization, its airborne concentration may not decline as sharply as infectivity. These findings indicate that BCoV RNA is more resilient than infectivity during airborne suspension, likely due to the RNA’s inherent stability even after partial virion damage or inactivation.

**Figure 4:**
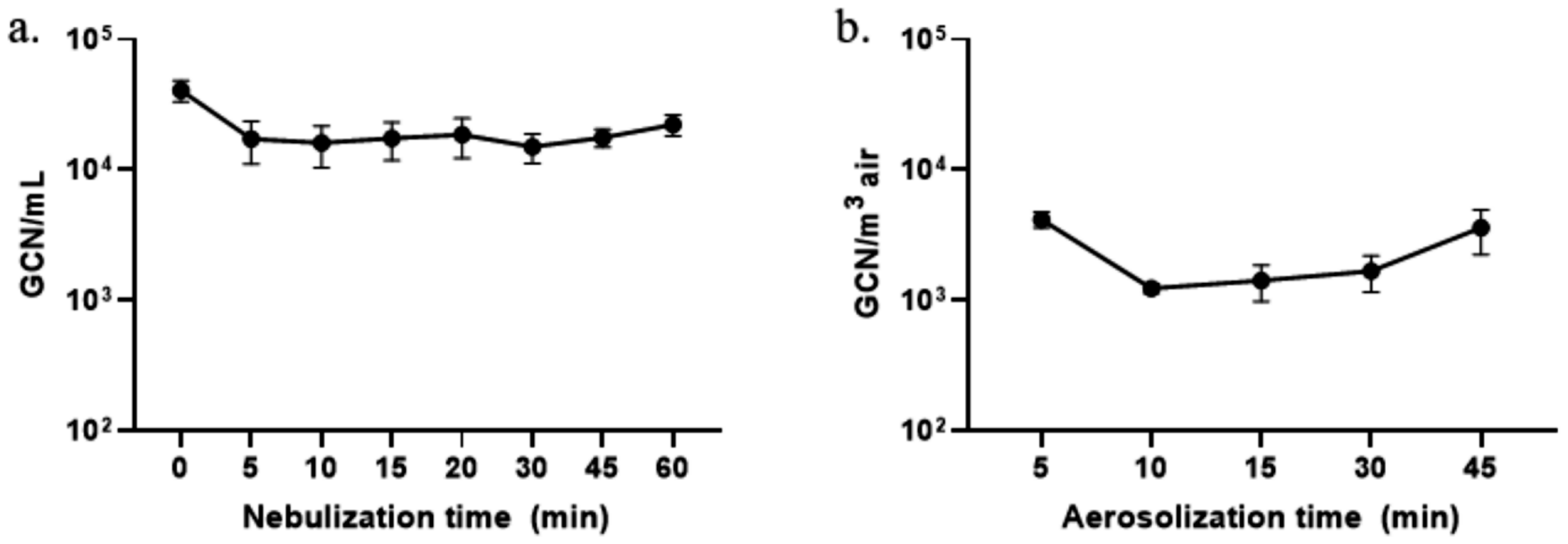
**(A)** BCoV GCN/mL for the virus suspension in the nebulizer after 0, 5, 10, 15, 20, 30, 45, and 60 min nebulization, and **(B)** BCoV GCN/m^3^ of air for the WWC-collected aerosols after 5, 10, 15, 30, and 45 min aerosolization along with air mixing.

To further explore the impact of nebulization time on BCoV RNA levels in liquid suspensions, BCoV GCN values were measured after 0 (stock), 5, 10, 15, 20, 30, 45, and 60 min of continuous nebulization (Figure 4A). A pronounced decrease in viral GCN was observed immediately after 5 min of nebulization, with levels dropping from 4.09 × 10^4^ GCN/mL to 1.73 × 10^4^ GCN/mL. However, beyond this initial drop, the viral GCNs remained relatively stable, fluctuating only slightly between 1.51 × 10^4^ GCN/mL and 2.23 × 10^4^ GCN/mL for the remainder of the 60-min nebulization. One-way ANOVA analysis did not find this variation to be statistically significant (*p* = 0.15), and *post hoc* comparisons between time points (*p* ≥ 0.26 for all comparisons) confirmed the absence of meaningful differences. This pattern supports the notion that most of the RNA loss occurs early during nebulization, possibly due to initial mechanical stress, and reaches a steady state thereafter. For WWC-collected BCoV aerosol samples with normalization to the stock, the highest (4.19 × 10^3^ GCN/m^3^ of air) and lowest (1.24 × 10^3^ GCN/m^3^ of air) viral RNA levels were observed at 5 min and 10 min of aerosolization, respectively (Figure 4B). Although a clear monotonic decrease in GCN with increasing aerosolization time was not observed, a moderate increase in RNA concentration was noted from 15 to 45 min. These differences were not statistically significant based on multiple comparison testing (*p* ≥ 0.18 for all comparisons), suggesting that RNA levels in the aerosols remained relatively stable and were not strongly affected by extended suspension times under the tested conditions.

**Figure 5:**
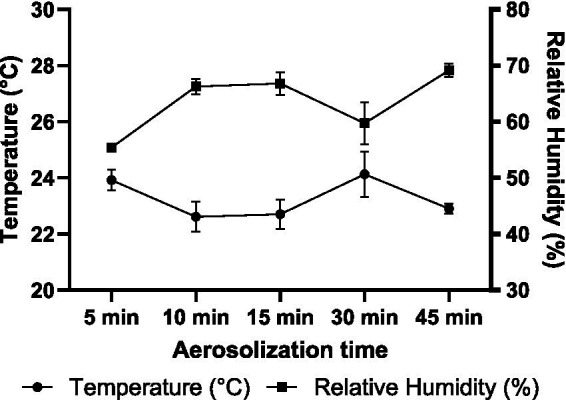
Average temperature and relative humidity in the testing chamber during the 5, 10, 15, 30, and 45 min aerosolization duration.

Environmental conditions within the testing chamber were monitored throughout the experiments, as temperature and relative humidity are known to influence both virus viability and aerosol sampling efficiency. Across the five aerosolization durations, temperature ranged from 22.6 and 24.1 °C, and the average relative humidity ranged from 55.4 to 69.1% (Figure 5). As expected, an inverse relationship between temperature and relative humidity was observed, consistent with the physics of air’s moisture-holding capacity. This occurs as warmer air can hold more water vapor before becoming saturated. Unless additional moisture is introduced, relative humidity decreases as temperature increases. Despite the continuous generation of aerosols contributing water vapor to the chamber, fluctuations in the temperature still drive the observed opposite trends in relative humidity. Kruskal–Wallis analysis showed a statistically significant overall difference in temperature across aerosolization durations (*p* = 0.01), but Dunn’s post-hoc tests found no significant pairwise differences (*p* ≥ 0.20 for all comparisons). This suggests that while temperature varied slightly over time, the differences between individual conditions were modest and unlikely to have biological relevance. On the other hand, relative humidity showed more variation and ANOVA revealed a significant overall difference (*p* = 0.0003). Dunn’s test identified a significant increase in relative humidity between the 5 min and 45 min time points (*p* = 0.02), while other comparisons were not significant (*p* ≥ 0.17), indicating that major changes were limited to select time point.

## Discussion

This study examined the infectivity and RNA stability of aerosolized BCoV across different durations of aerosolization in a mechanically mixed chamber environment. Our results demonstrated that while the nebulization process caused minimal loss of viral infectivity in the liquid suspension, prolonged aerosol residence under mechanical mixing markedly reduced infectious titers of the viruses. In contrast, viral RNA remained comparatively stable throughout the aerosolization period, even as infectivity declined. The observation that pre- and post-nebulized BCoV suspensions exhibited similar infectious titers suggests that the shear and impaction forces inside the Collison nebulizer do not substantially impair virion infectivity. While mechanical shear during the nebulization could induce virion alterations that are not immediately reflected as infectivity loss, prior studies have reported high preservation (over 80%) of viability following Collison nebulization for other enveloped viruses, including influenza A viruses, SARS-CoV-2, and PRD1 bacteriophage (Baig et al., 2022; Niazi et al., 2021; Paton et al., 2021). Nevertheless, sublethal effects may exist that could influence host cell entry or replication, and future studies should include analysis of the structural integrity of the virions. On the other hand, our results revealed a consistent reduction in viral RNA immediately following nebulization in the nebulizer reservoir across all aerosolization durations. This early RNA loss that was most obvious within the first five minutes of aerosol generation likely reflects an initial effect of mechanical stress, including shear or impaction, as well as possible adsorption to internal surfaces (Niazi et al., 2021). Notably, after this rapid initial decrease, RNA levels in the nebulizer reservoir stabilize, fluctuating only modestly between 1.51 × 10^4^ GCN/mL and 2.23 × 10^4^ GCN/mL over 60 min. One potential explanation is that once a subset of genomes is degraded or adsorbed, the remaining RNA achieves a dynamic equilibrium, perhaps protected within intact virions or by equilibrium between genome release and further loss. Similar patterns have been observed in a study using a Collison nebulizer to generate influenza aerosols, where no loss of viable virus or viral RNA was detected in the nebulizer reservoir post-nebulization while airborne titers declined more substantially as airborne viral RNA remained stable (Brown et al., 2015). Another study nebulized transmissible gastroenteritis virus (TGEV) using both Collison and medical nebulizers at varying pressures, and found no significant reduction (less than 10%) in virus titer in the nebulizer suspension before and after nebulization, which is consistent with our findings that the mechanical process of nebulization does not inherently reduce viral infectivity in suspension (Kim et al., 2007). Similar minimal loss of virus viability was found by Hermann et al. after 55 min nebulization of enveloped porcine reproductive and respiratory syndrome virus (PRRSV) (Hermann et al., 2006). These studies along with ours using different types of enveloped viruses suggest that while nebulization generates small respirable droplets suitable for experimental aerosolization, the shear and impaction forces involved in the 6-jet Collison nebulizer do not necessarily compromise the virus envelope or capsid structure. Once airborne, however, BCoV infectivity significantly declined over time from 5 to 45 min of aerosolization with mechanical air mixing. This decline aligns with prior studies on aerosols of various types of coronaviruses including SARS-CoV-2, SARS-CoV, and mouse hepatitis virus (MHV) that have demonstrated similar time-dependent reductions in virus titers within controlled environments (Doremalen et al., 2020; Oswin et al., 2021). Other than the mechanical forces imposed from air mixing, the loss of infectivity in aerosols can also be attributable to a combination of physical and environmental stressors such as droplet evaporation, increased chance of impaction damage, desiccation, and degradation (Brown et al., 2015; Rezaei and Netz, 2021). Despite the markable loss of infectivity at longer aerosolization time, viral RNA levels measured by qRT-PCR remained relatively constant, fluctuating within a narrow range. This disparity suggests that the viral genome is more resilient to airborne degradation than the infectious virion itself, a pattern observed in influenza viruses as well (Brown et al., 2015). A number of studies has shown that RNA from SARS-CoV-2 can persist on surfaces long after viable virus is undetectable (Paton et al., 2021; Puhach et al., 2023; Kasloff et al., 2021; Yamagishi et al., 2020). Our results show that this higher resilience of viral RNA is also relevant in BCoV aerosols, highlighting the potential for molecular methods to overestimate infectious risk if not interpreted in conjunction with culture-based assays. These findings reinforce the importance of pairing qRT-PCR detection with infectivity assays to fully characterize airborne virus viability, particularly in environmental or clinical surveillance contexts.

Environmental conditions within the chamber remained broadly stable yet displayed measurable trends over the course of aerosolization. Temperatures recorded during the five sampling intervals ranged narrowly from 22.6 °C to 24.1 °C, whereas relative humidity increased from 55.4% at 5 min to 69.1% at 45 min. The inverse relationship between temperature and relative humidity reflects the physics of air’s moisture-holding capacity, yet in our closed system, continuous aerosol generation offset some of this effect by introducing additional moisture. The small temperature changes observed over the aerosolization period were statistically detectable but unlikely to have meaningful biological impact. In contrast, relative humidity exhibited a significant overall increase, especially between the 5 min and 45 min time point. Previous studies reported that relative humidity (20% vs. 70%) moderately affected the infectivity of SARS-CoV-2 aerosols, though to a lesser extent than temperature or UV (Dabisch et al., 2021). A model using modified Wells-Riley equations revealed virus-specific responses to relative humidity - the infection risk of airborne influenza increases with relative humidity over 20 to 80%, while SARS-CoV-2 aerosols showed a non-monotonic pattern, having the highest infection risk at 53% relative humidity and declining at both lower and higher levels (Aganovic et al., 2022). Another controlled experiment has also shown that the impact of relative humidity on airborne SARS-CoV-2 infectivity is complex and dependent on the suspension matrix used (Smither et al., 2020). Consistent trends have also been reported for airborne influenza A viruses, where Motos et al. observed that viruses aerosolized in saline remained infectious for the longest time at low relative humidity (<30%) but exhibited rapid infectivity loss at elevated relative humidity (40 to 70%) (Motos et al., 2024). In our study, the gradual increase in relative humidity may have contributed to BCoV inactivation in aerosols but was likely acted jointly with mechanical mixing, evaporative stress, and airborne residence time rather than as an isolated driver of inactivation. Moreover, the relative humidity levels in our tests (55 to 70%) fall within the mid-range evaluated in previous studies and are less likely to exert a pronounced effect on viral stability.

While the findings in this present study suggest that coronaviruses infectivity, using BCoV as a surrogate, declines during prolonged airborne suspension, several limitations should be acknowledged. First, the study was conducted under a specific set of environmental conditions, including a narrow temperature range (22.6 °C to 24.1 °C) and moderate relative humidity (55 to 70%), which may not capture the full spectrum of environmental variability encountered in real-world settings. Second, the use of a sealed, continuously mixed chamber may not fully represent natural airflow dynamics or deposition processes in occupied indoor environments. The propeller mixed chamber does not replicate the linear airflow profiles of HVAC systems. Rather, it provides a controlled environment to examine mechanical mixing and airborne residence under recirculating conditions commonly encountered in occupied indoor spaces with fans or localized ventilation. Prior APS measurements and CFD modeling under identical conditions demonstrated homogeneous aerosol dispersion and stable particle residence times, supporting the interpretation that infectivity loss reflects cumulative airborne stress rather than uneven deposition or selective loss of larger droplets (Baig et al., 2022; Smith et al., 2025). Third, the use of a single virus surrogate BCoV limits the generalizability of findings to other coronaviruses with different structural or physicochemical properties. Lastly, the temporal resolution of aerosol sampling was restricted to discrete time points, which may overlook more transient changes in virus viability. While the nebulized medium did not include complex respiratory fluids such as saliva or mucus, the use of 10x diluted PBS containing 0.08% NaCl approximates the NaCl content of saliva (0.017–0.123%). These limitations highlight the need for future studies that incorporate respiratory matrices, multiple virus strains, and additional environmental variables such as light exposure and air pollutants to enhance our understanding.

## Conclusion

In this study, we investigated how aerosolization and airborne suspension affect coronavirus infectivity and RNA persistence under controlled mixed-air conditions, using BCoV as a surrogate. While the nebulization showed minimal effect on the viral infectivity of the virus suspension in the reservoir, continuous air mixing and prolonged residence time led to a progressive loss of virus infectivity in the aerosols. In contrast, viral RNA levels in both reservoir and WWC-collected aerosols remained comparatively stable, underscoring the greater resilience of genomic material and the importance of combining molecular and culture-based assays for accurate risk assessment. Moderate increases in relative humidity and minimal temperature fluctuations in the present study likely played a secondary role in virus inactivation relative to mechanical shear and evaporative stress. These findings advance our understanding of airborne coronavirus persistence and highlight the need for future studies that incorporate broader environmental conditions and human-relevant coronavirus surrogates to inform mitigation strategies for indoor airborne transmission.

## Data Availability

The original contributions presented in the study are included in the article/Supplementary material, further inquiries can be directed to the corresponding author/s.

## References

[ref1] Aboubakr H. A. Sharafeldin T. A. Goyal S. M. (2021). Stability of SARS-CoV-2 and other coronaviruses in the environment and on common touch surfaces and the influence of climatic conditions: a review. Transbound. Emerg. Dis. 68, 296–312. doi: 10.1111/tbed.13707, 32603505 PMC7361302

[ref2] Aganovic A. Bi Y. Cao G. Kurnitski J. Wargocki P. (2022). Modeling the impact of indoor relative humidity on the infection risk of five respiratory airborne viruses. Sci. Rep. 12:11481. doi: 10.1038/s41598-022-15703-8, 35798789 PMC9261129

[ref3] Baig T. A. Zhang M. Smith B. L. King M. D. (2022). Environmental effects on viable virus transport and resuspension in ventilation airflow. Viruses 14:616. doi: 10.3390/v14030616 35337023 PMC8950092

[ref4] Bellini W.J. (2003). SARS-CoV specific RT-PCR primers. Available online at: https://www.who.int/publications/m/item/sars-cov-specific-rt-pcr-primers

[ref5] Biryukov J. Boydston J. A. Dunning R. A. Yeager J. J. Wood S. Reese A. L. . (2020). Increasing temperature and relative humidity accelerates inactivation of SARS-CoV-2 on surfaces. mSphere 5:e00441-20. doi: 10.1128/msphere.00441-20 32611701 PMC7333574

[ref6] Brown J. R. Tang J. W. Pankhurst L. Klein N. Gant V. Lai K. M. . (2015). Influenza virus survival in aerosols and estimates of viable virus loss resulting from aerosolization and air-sampling. J. Hosp. Infect. 91, 278–281. doi: 10.1016/j.jhin.2015.08.004, 26412395

[ref7] Cantu J. C. Butterworth J. W. Mylacraine K. S. Ibey B. L. Gamboa B. M. Johnson L. R. . (2023). Evaluation of inactivation of bovine coronavirus by low-level radiofrequency irradiation. Sci. Rep. 13:9800. doi: 10.1038/s41598-023-36887-7, 37328590 PMC10275941

[ref8] Casanova L. M. Jeon S. Rutala W. A. Weber D. J. Sobsey M. D. (2010). Effects of air temperature and relative humidity on coronavirus survival on surfaces. Appl. Environ. Microbiol. 76, 2712–2717. doi: 10.1128/AEM.02291-09, 20228108 PMC2863430

[ref9] Chan K. H. Peiris J. S. Lam S. Y. Poon L. L. Yuen K. Y. Seto W. H. (2011). The effects of temperature and relative humidity on the viability of the SARS coronavirus. Adv Virol. 2011:734690. doi: 10.1155/2011/734690 22312351 PMC3265313

[ref10] Dabisch P. Schuit M. Herzog A. Beck K. Wood S. Krause M. . (2021). The influence of temperature, humidity, and simulated sunlight on the infectivity of SARS-CoV-2 in aerosols. Aerosol Sci. Technol. 55, 142–153. doi: 10.1080/02786826.2020.1829536 38077296 PMC10698725

[ref11] Doremalen N. V. Bushmaker T. Morris D. H. Holbrook M. G. Gamble A. Williamson B. N. . (2020). Aerosol and surface stability of SARS-CoV-2 as compared with SARS-CoV-1. N. Engl. J. Med. 382, 1564–1567. doi: 10.1056/NEJMc2004973 32182409 PMC7121658

[ref12] Farhud D. Azari M. Mehrabi A. (2022). The history of Corona virus: from Neanderthals to the present time: a brief review. Iran. J. Public Health 51, 531–534. doi: 10.18502/ijph.v51i3.8928 35865048 PMC9276611

[ref13] Graham K. E. Loeb S. K. Wolfe M. K. Catoe D. Sinnott-Armstrong N. Kim S. . (2021). SARS-CoV-2 RNA in wastewater settled solids is associated with COVID-19 cases in a large urban Sewershed. Environ. Sci. Technol. 55, 488–498. doi: 10.1021/acs.est.0c06191, 33283515

[ref14] Guérin-Rechdaoui S. Bize A. Levesque-Ninio C. Janvier A. Lacroix C. Le-Brizoual F. . (2022). Fate of SARS-CoV-2 coronavirus in wastewater treatment sludge during storage and thermophilic anaerobic digestion. Environ. Res. 214:114057. doi: 10.1016/j.envres.2022.114057 35995225 PMC9391084

[ref15] Haig C. W. Mackay W. G. Walker J. T. Williams C. (2016). Bioaerosol sampling: sampling mechanisms, bioefficiency and field studies. J. Hosp. Infect. 93, 242–255. doi: 10.1016/j.jhin.2016.03.017, 27112048 PMC7124364

[ref16] Harapan H. Johar E. Maroef C. N. Sriyani I. Y. Iqhrammullah M. Kusuma H. I. . (2022). Effect of elevated temperature on SARS-CoV-2 viability. F1000Res 11:403. doi: 10.12688/f1000research.110305.2 37745627 PMC10517306

[ref17] Hermann J. R. Hoff S. J. Yoon K. J. Burkhardt A. C. Evans R. B. Zimmerman J. J. (2006). Optimization of a sampling system for recovery and detection of airborne porcine reproductive and respiratory syndrome virus and swine influenza virus. Appl. Environ. Microbiol. 72, 4811–4818. doi: 10.1128/AEM.00472-06, 16820475 PMC1489351

[ref18] Herstein J. J. Degarege A. Stover D. Austin C. Schwedhelm M. M. Lawler J. V. . (2021). Characteristics of SARS-CoV-2 transmission among meat processing Workers in Nebraska, USA, and effectiveness of risk mitigation measures. Emerg. Infect. Dis. 27, 1032–1038. doi: 10.3201/eid2704.204800 33591249 PMC8007314

[ref19] Ijaz M. K. Brunner A. H. Sattar S. A. Nair R. C. Johnson-Lussenburg C. M. (1985). Survival characteristics of airborne human coronavirus 229E. J. Gen. Virol. 66, 2743–2748. doi: 10.1099/0022-1317-66-12-2743 2999318

[ref20] Islam A. Ferdous J. Islam S. Sayeed M. A. Dutta Choudhury S. Saha O. . (2021). Evolutionary dynamics and epidemiology of endemic and emerging coronaviruses in humans, domestic animals, and wildlife. Viruses 13:1908. doi: 10.3390/v13101908 34696338 PMC8537103

[ref21] Kasloff S. B. Leung A. Strong J. E. Funk D. Cutts T. (2021). Stability of SARS-CoV-2 on critical personal protective equipment. Sci. Rep. 11:984. doi: 10.1038/s41598-020-80098-3 33441775 PMC7806900

[ref22] Kim S. W. Ramakrishnan M. A. Raynor P. C. Goyal S. M. (2007). Effects of humidity and other factors on the generation and sampling of a coronavirus aerosol. Aerobiologia 23, 239–248. doi: 10.1007/s10453-007-9068-9, 32214623 PMC7087841

[ref23] Kimbrell B. Huang J. Fraser A. Jiang X. (2024). Efficacy of three antimicrobials against two SARS-COV-2 surrogates, bovine coronavirus and human coronavirus OC43, on hard or soft nonporous materials. J. Food Prot. 87:100316. doi: 10.1016/j.jfp.2024.100316, 38878900

[ref24] King M. D. McFarland A. R. (2012). Bioaerosol sampling with a Wetted Wall cyclone: cell Culturability and DNA integrity of Escherichia coli Bacteria. Aerosol Sci. Technol. 46, 82–93. doi: 10.1080/02786826.2011.605400

[ref25] LaTurner Z. W. Zong D. M. Kalvapalle P. Gamas K. R. Terwilliger A. Crosby T. . (2021). Evaluating recovery, cost, and throughput of different concentration methods for SARS-CoV-2 wastewater-based epidemiology. Water Res. 197:117043. doi: 10.1016/j.watres.2021.117043 33784608 PMC7957301

[ref26] Lauterbach S. E. Wright C. M. Zentkovich M. M. Nelson S. W. Lorbach J. N. Bliss N. T. . (2018). Detection of influenza a virus from agricultural fair environment: air and surfaces. Prev. Vet. Med. 153, 24–29. doi: 10.1016/j.prevetmed.2018.02.019, 29653731 PMC8611410

[ref27] McNeill V. F. (2022). Airborne transmission of SARS-CoV-2: evidence and implications for engineering controls. Annual Review Chemical Biomolecular Engineering 13, 123–140. doi: 10.1146/annurev-chembioeng-092220-111631 35300517

[ref28] Meng F. King M. D. Hassan Y. A. Ugaz V. M. (2014). Localized fluorescent complexation enables rapid monitoring of airborne nanoparticles. Environ. Sci. Nano. 1, 358–366. doi: 10.1039/C4EN00017J

[ref29] Motos G. Schaub A. David S. C. Costa L. Terrettaz C. Kaltsonoudis C. . (2024). Dependence of aerosol-borne influenza a virus infectivity on relative humidity and aerosol composition. Front. Microbiol. 15:1484992. doi: 10.3389/fmicb.2024.1484992 39479211 PMC11521868

[ref30] Niazi S. Philp L. K. Spann K. Johnson G. R. (2021). Utility of three nebulizers in investigating the infectivity of airborne viruses. Appl. Environ. Microbiol. 87:e0049721. doi: 10.1128/AEM.00497-21 34085856 PMC8373236

[ref31] Oswin H. P. Haddrella A. E. Otero-Fernandez M. Cogan T. A. Mann J. F. S. Morley C. H. . (2021). Measuring stability of virus in aerosols under varying environmental conditions. Aerosol Sci. Technol. 55, 1315–1320. doi: 10.1080/02786826.2021.1976718

[ref32] Paton S. Spencer A. Garratt I. Thompson K. A. Dinesh I. Aranega-Bou P. . (2021). Persistence of severe acute respiratory syndrome coronavirus 2 (SARS-CoV-2) virus and viral RNA in relation to surface type and contamination concentration. Appl. Environ. Microbiol. 87:e0052621. doi: 10.1128/AEM.00526-21 33962986 PMC8231718

[ref33] Peeri N. C. Shrestha N. Rahman M. S. Zaki R. Tan Z. Bibi S. . (2020). The SARS, MERS and novel coronavirus (COVID-19) epidemics, the newest and biggest global health threats: what lessons have we learned? Int. J. Epidemiol. 49, 717–726. doi: 10.1093/ije/dyaa033, 32086938 PMC7197734

[ref34] Pokora R. Kutschbach S. Weigl M. Braun D. Epple A. Lorenz E. . (2021). Investigation of superspreading COVID-19 outbreak events in meat and poultry processing plants in Germany: a cross-sectional study. PLoS One 16:e0242456. doi: 10.1371/journal.pone.0242456, 34111143 PMC8191887

[ref35] Puhach O. Meyer B. Eckerle I. (2023). SARS-CoV-2 viral load and shedding kinetics. Nat. Rev. Microbiol. 21, 147–161. doi: 10.1038/s41579-022-00822-w 36460930 PMC9716513

[ref36] Pyankov O. V. Bodnev S. A. Pyankova O. G. Agranovski I. E. (2018). Survival of aerosolized coronavirus in the ambient air. J. Aerosol Sci. 115, 158–163. doi: 10.1016/j.jaerosci.2017.09.009 32226116 PMC7094304

[ref37] Redmann R. K. Kaushal D. Golden N. Threeton B. Killeen S. Z. Kuehl P. J. . (2022). Particle dynamics and bioaerosol viability of aerosolized Bacillus Calmette-Guérin vaccine using jet and vibrating mesh clinical nebulizers. J. Aerosol Med. Pulm. Drug Deliv. 35, 50–56. doi: 10.1089/jamp.2021.0030, 34619040 PMC8867098

[ref38] Reed L. J. Muench H. (1938). A simple method of estimating fifty per cent endpoints12. Am. J. Epidemiol. 27, 493–497.

[ref39] Ren S. Y. Wang W. B. Hao Y. G. Zhang H. R. Wang Z. C. Chen Y. L. . (2020). Stability and infectivity of coronaviruses in inanimate environments. World J. Clin. Cases 8, 1391–1399. doi: 10.12998/wjcc.v8.i8.1391, 32368532 PMC7190947

[ref40] Rezaei M. Netz R. R. (2021). Airborne virus transmission via respiratory droplets: effects of droplet evaporation and sedimentation. Curr. Opin. Colloid Interface Sci. 55:101471. doi: 10.1016/j.cocis.2021.101471 34093064 PMC8164513

[ref41] Riddell S. Goldie S. Hill A. Eagles D. Drew T. W. (2020). The effect of temperature on persistence of SARS-CoV-2 on common surfaces. Virol. J. 17:145. doi: 10.1186/s12985-020-01418-7, 33028356 PMC7538848

[ref42] Schuit M. Ratnesar-Shumate S. Yolitz J. Williams G. Weaver W. Green B. . (2020). Airborne SARS-CoV-2 is rapidly inactivated by simulated sunlight. J. Infect. Dis. 222, 564–571. doi: 10.1093/infdis/jiaa334 32525979 PMC7313838

[ref43] Sharma A. Preece B. Swann H. Fan X. McKenney R. Ori-McKenney K. . (2021). Structural stability of SARS-CoV-2 virus like particles degrades with temperature. Biochem. Biophys. Res. Commun. 534, 343–346. doi: 10.1016/j.bbrc.2020.11.080, 33272571 PMC7699159

[ref44] Smith B. L. King M. D. (2022). Aerosolization triggers immediate antibiotic resistance in bacteria. J. Aerosol Sci. 164:106017. doi: 10.1016/j.jaerosci.2022.106017

[ref45] Smith B. L. King M. D. (2023). Quiescence of Escherichia coli aerosols to survive mechanical stress during high-velocity collection. Microorganisms 11:647. doi: 10.3390/microorganisms11030647 36985220 PMC10058004

[ref46] Smith B. L. Zhang M. King M. D. (2025). Airborne Escherichia coli bacteria biosynthesize lipids in response to aerosolization stress. Sci. Rep. 15:2349. doi: 10.1038/s41598-025-86562-2, 39833243 PMC11746921

[ref47] Smith B. L. Zhang M. Kumar S. King M. D. (2025). Aerosolization affects Bacillus globigii vegetative cell and spore behaviors. Microorganisms 13:2532. doi: 10.3390/microorganisms13112532 41304218 PMC12654006

[ref48] Smither S. J. Eastaugh L. S. Findlay J. S. Lever M. S. (2020). Experimental aerosol survival of SARS-CoV-2 in artificial saliva and tissue culture media at medium and high humidity. Emerg Microbes Infect 9, 1415–1417. doi: 10.1080/22221751.2020.1777906, 32496967 PMC7473326

[ref49] Thornton G. M. Kroeker E. Fleck B. A. Zhong L. Hartling L. (2022). The impact of heating, ventilation, and air-conditioning design features on the transmission of viruses, including SARS-CoV-2: overview of reviews. Interact J Med Res 11:e37232. doi: 10.2196/37232 36343208 PMC9823592

[ref50] van Doremalen N. Bushmaker T. Munster V. J. (2013). Stability of Middle East respiratory syndrome coronavirus (MERS-CoV) under different environmental conditions. Euro Surveill. 18:20590. doi: 10.2807/1560-7917.ES2013.18.38.20590 24084338

[ref51] Watanabe M. Ohnishi T. Arai S. Kawakami T. Hayashi K. Ohya K. . (2022). Survival of SARS-CoV-2 and bovine coronavirus on common surfaces of living environments. Sci. Rep. 12:10624. doi: 10.1038/s41598-022-14552-9, 35739204 PMC9218704

[ref52] Wei Y. Dong Z. Fan W. Xu K. Tang S. Wang Y. . (2022). A narrative review on the role of temperature and humidity in COVID-19: transmission, persistence, and epidemiological evidence. Eco Environ Health 1, 73–85. doi: 10.1016/j.eehl.2022.04.006, 38013745 PMC9181277

[ref53] Yamagishi T. Ohnishi M. Matsunaga N. Kakimoto K. Kamiya H. Okamoto K. . (2020). Environmental sampling for severe acute respiratory syndrome coronavirus 2 during a COVID-19 outbreak on the diamond princess cruise ship. J. Infect. Dis. 222, 1098–1102. doi: 10.1093/infdis/jiaa437 32691828 PMC7454703

[ref54] Zhang M. King M. D. (2023). Temporal variation of SARS-CoV-2 levels in wastewater from a meat processing plant. Microorganisms 11:174. doi: 10.3390/microorganisms11010174 36677465 PMC9864470

[ref55] Zhang M. Smith B. L. Wilsey R. N. Koch T. N. M. Dohanich E. McShane P. J. . (2025). Culture and real-time quantitative PCR to detect environmental nontuberculous mycobacteria in a clinical care center. Total Environment Microbiology 1:100016. doi: 10.1016/j.temicr.2025.100016 41716950 PMC12916027

[ref56] Zhang M. Wang H. Foster E. R. Nikolov Z. L. Fernando S. D. King M. D. (2022). Binding behavior of spike protein and receptor binding domain of the SARS-CoV-2 virus at different environmental conditions. Sci. Rep. 12:789. doi: 10.1038/s41598-021-04673-y, 35039570 PMC8763896

