# Supplementary material for: Aerosolization effects on coronavirus infectivity
Source: Front Microbiol. 2026 Feb 20;17:1764341. doi: 10.3389/fmicb.2026.1764341 (PMC12963325; doi:10.3389/fmicb.2026.1764341)
Supplement: Supplementary file 1 [file Supplementary_file_1.docx]

**Supplementary Materials**

**Figure S1.** qRT-PCR standard curve for BCoV with corresponding regression equation and R^2^ values. The amplification efficiency was 78.3%. The LoD was 289 GCN/mL.
